# Do Personality Features Influence Our Intuitions of the Mind-Body Problem? A Pilot Study

**DOI:** 10.3389/fpsyg.2018.01219

**Published:** 2018-07-20

**Authors:** Marek Havlík, Karolína Mladá, Iveta Fajnerová, Jiří Horáček

**Affiliations:** ^1^National Institute of Mental Health, Klecany, Czechia; ^2^Third Faculty of Medicine, Charles University, Prague, Czechia

**Keywords:** mind-body problem, experimental philosophy, intuitions of reduction, temperament and character inventory, self-transcendence

## Abstract

The elusive relationship between the mental domain and the physical brain, known as the mind-body problem, is still a hot topic of discussion among philosophers and neuroscientists. Rather than solving this problem, our pilot study addresses the question as to whether personality features could influence intuitions of the mind-body problem, or more precisely, whether it is possible to identify a person’s intuitive inclinations toward dualism or materialism and their inclinations toward reduction of the mind to the brain. For the purposes of this pilot study, we developed a questionnaire, which employed several theories of analytic philosophy of the mind, in order to determine which category the participants would belong to. These main categories were dualism, non-reductive materialism and reductive materialism. To test whether personality features affect preferences for these categories, the participants were investigated by Cloninger’s Temperament and Character Inventory (TCI). We found significant differences in the self-transcendence dimension of the TCI between participants who were evaluated as dualists and those who were assessed as reductive materialists. Our data show that the personality dimension of self-transcendence correlates with intuitive inclination toward reductive materialism or dualism. In addition, our results suggest that ideas, theories, and hypothetical solutions of the mind-body problem and possibly even conclusions, acceptance, and disputations of thought experiments of philosophy of the mind can be biased by personality traits. This fact should be taken into account in future discussions of the philosophy of the mind and may also be important for empirical research and an empirical understanding of the mind.

## Introduction

The mind-body problem represents one of the most enigmatic and open questions in the fields of philosophy and neuroscience. It refers to the relationship between mental content and its physical (brain) substrate. There are many possible ways to approach this problem, varying from the various forms of dualism to the various forms of monism. The mind-body problem has been considered as the main problem of the philosophy of the mind since Descartes (1641) formulated the crucial difference between *material substance (res extensa)* and *mental substance (res cogitans)*, thus creating substance dualism. Three centuries later, Gilber Ryle, considered by some to be one of the fathers of modern philosophy of the mind, principally criticized Descartes’ (1641) distinction and branded it as a *category* *mistake* (Ryle, 1949). Even though 20th century philosophy of the mind was dominated by materialistic theories, dualism still found its way to the discussions of the nature of consciousness, where it became an implicit tool in modern modal and epistemic arguments for the irreducibility of consciousness (e.g., Nagel, 1974; Jackson, 1982; Levine, 1983; Chalmers, 1996).

It is common for two equally well-educated philosophers to have completely different beliefs about the mind, consciousness or qualia (subjective qualitative aspects of conscious experience). However, what is much more interesting is that even some empirical scientists, who would be expected to incline toward reductionist views, incline toward dualism. For example, Charles S. Sherrington, Wilder Penfield, and John C. Eccles, who are considered among the most renowned neuroscientists of the 20th century, openly supported the dualistic view of the mind and brain. Despite the fact that the explicit dualistic position is not congruent with the modern empirical science, it still persists. It shows two essential aspects of the mind-body problem. First, it is methodologically extremely problematic and not empirically resolvable, since the empirical approaches are typically not causative but only correlative in nature. Second, the fact that people (including philosophers and neuroscientists) differ in intuitions about the relation of mind and body indicates the existence of underlying psychological factors that are responsible for the inter-individual differences.

Such ideas are not entirely new. Several thinkers have proposed that different psychological processes are the basis for various forms of beliefs, scientific concepts, forms of knowledge and different scientific methods. For example, Jung (1921/1971) considered that individual differences, such as extraversion and introversion, stay behind the various philosophical antinomies (Lockean philosophy vs. Liebnizian philosophy) and play an important role in their positive or negative reception. Coan (1979), based on the data from the Theoretical Orientation Survey, considered various psychological type processes to be the driving force behind choosing and preferring a specific theoretical and methodological position in psychology. Psychological processes can also influence the choice between mechanistic or vitalistic interpretations of reality and even stay behind the emphasis on the different psychological phenomena, which define individual paradigms of psychology (e.g., behaviorism, structuralism, gestalt psychology, etc.) and their methods (e.g., emphasis on introspection) (Tobacyk, 2003). According to Barušs (1990, 2008), the inter-individual differences in beliefs about consciousness and reality (materialistic beliefs vs. transcendentally based beliefs) are the main reason for the confusions of the notion of consciousness.

In recent years, inter-individual differences have become the subject of a new philosophical discipline, experimental philosophy. Experimental philosophy, aspiring to enrich the ‘armchair philosophy’ by an empirical approach (Knobe, 2004, 2007), is primarily focused on the study of genuine intuitions of individuals who are not trained in philosophy. According to Knobe and Nichols (2008), identifying underlying psychological processes, which are responsible for inter-individual differences in intuitions, is one of the goals of experimental philosophy. This new philosophical approach typically addresses topics such as determinism and moral judgments (Nichols and Knobe, 2007), intentional actions (Knobe, 2003), cultural diversity (Machery et al., 2015), and intuitions about folk psychology and phenomenal consciousness (Robbins and Jack, 2006; Gray et al., 2007; Haslam et al., 2008; Knobe and Prinz, 2008; Huebner, 2010).

We assume that every person takes some intuitive position toward the relationship between mental phenomena and physical processes of the brain. However, to our knowledge there is no systematic study that would try to identify what possibly drives the intuitions of the mind-body problem and intuitions of reduction of mental to brain processes.

In this pilot study, we aimed to identify psychological personality traits that correlate with a personal inclination toward three different approaches in analytic philosophy of the mind. The first is dualism, the idea that the immaterial mind (immaterial and independent soul) occupies the material body, which represents a complex machine but nothing more. The second is non-reductive materialism, whose supporters claim that the mind is created by the materialistic brain; however, mental states do exist and cannot be reduced to simple physiological processes. Finally, there is reductive materialism, which says that the mind is nothing more than brain processes and can (or will) be completely explained by them.

For the purpose of our study, we developed a Mind-Body Questionnaire (MBQ), which measures the participant’s degree of dualistic or reductionist intuitions about the mind and body. To measure the various dimensions of personality, we employed the Temperament and Character Inventory (TCI) (Cloninger et al., 1993, 1994). Cloninger’s (1986) psycho-biologically based model of personality is a well-validated personality questionnaire, which operates with seven personality dimensions covering two main concepts of personality: temperament and character (Cloninger et al., 1994; Zuckerman and Cloninger, 1996). Temperament, also called emotionality (Cloninger et al., 1998), consists of four dimensions – *novelty seeking*, *harm avoidance*, *reward dependence*, *persistence* – and represents “biologically based components of personality, which are independently heritable, manifest early in life, and involve pre-conceptual biases in perceptual memory and habit formation” (Cloninger et al., 1993, p. 975). Character consists of dimensions of *self-directedness*, *cooperativeness*, and *self-transcendence* and represents the consciously learned component of personality. Character is the way people relate to themselves and to others and reflects individual differences based on experience and sociocultural learning with foresight about the long-term consequences of choices (Cloninger et al., 1993; MacDonald et al., 1995; Josefsson et al., 2013). The recent large cohort family studies clearly support the psychobiological theory of temperament and character but suggest that both domains share equally large genetic influences (Gillespie et al., 2003; Yang et al., 2015).

In addition to the TCI, there are other personality questionnaires frequently used for research purposes such as the Eysenck and Eysenck’s (1975, 1991) Questionnaire or the NEO Five-Factor Inventory (NEO-FFI, Costa and McCrae, 1992). We have chosen Cloninger’s TCI approach for this study for several reasons. First, this personality typology is based on the assumption that independent TCI dimensions have a characteristic neurobiological substrate and these associations have been repeatedly confirmed by genetic and neuroimaging studies (Balestri et al., 2014; Leurquin-Sterk et al., 2016). Second, the clear distinction between “temperament” and “character” enables us to test if the intuitions about the mind-body problem are influenced by emotional (temperament) or cognitive (character) processes. Third, the combination of TCI personality dimensions exerts sufficient explanatory power for complex cognitive attitudes and beliefs as well as for long-term choices in life (Eley et al., 2015; Saarinen et al., 2018). Last, the TCI showed a considerable overlap with the NEO-FFI, so its results could be related to this alternative model to some extent (De Fruyt et al., 2000).

The main goal of this pilot study was to test whether personality *features* are associated with individual intuitions about the relationship of the mind and body and reduction of the mind to physical brain processes. Our *a priori* hypothesis was that the character dimensions (foremost self-transcendence, ST) are more strongly linked to such questions than the dimensions of temperament. Specifically, we expected that ST would be significantly more expressed in dualists than in reductive materialists, and would correlate with the dualistic position.

The expected association between ST and the dualistic position is non-trivial because ST not only referred to spirituality or religiosity but also related to all aspects of the sense of transcendence. Individuals with a high ST score regard themselves as integral parts of the universe (Cloninger et al., 1993, 1998; Farmer and Goldberg, 2008).

Furthermore, we also tested two secondary hypotheses that most of the participants would incline toward *non-reductive materialism*. We expected that most people would incline toward materialistic views; however, if it comes to mind, people would prefer non-reductive positions rather than ruthless reductive perspectives, because reductive positions include the reduction of the most intimate mental phenomena, such as self or volition. Finally, precisely because of these reasons we assumed that the most agreeable theory would be *Emergentism* (e.g., Searle, 1984), which considers the mind as a non-reductive higher property of the materialistic system (the brain).

## Materials and Methods

### Procedure

For the purposes of this pilot study, we developed a three-part online questionnaire. The first part was aimed at *Demography*, the second part was the Mind-Body Questionnaire (MBQ) and for the third part we utilized Cloninger’s TCI. This study was approved by the Ethics Committee of National Institute of Mental Health, Czechia. All data were collected with the informed consent of each participant and all of the participants provided consent indicating they understood the terms of involvement in the study.

### Participants

Participants with various levels of education, employment, age, and other characteristics were included in the study. Of the 116 participants who provided their demographic information and answered all of the items of the MBQ, 94 also completed the TCI. In the demographic part of the questionnaire, the participants were asked about their age, gender, marital status, profession, highest attained level of education, interest in philosophy, interest in science, interest in spirituality and esotericism, and religious beliefs.

### Mind-Body Questionnaire

The MBQ developed for the purpose of this study consists of 10 specific theories on the mind-body problem of the philosophy of the mind (see **Table 4**, for detailed characteristics of all theories see Appendix 1). The core argument of each mind-body theory was captured using short sentences, while trying to be as brief and clear as possible. We chose theories related to dualism, non-reductive materialism, and reductive materialism, which we considered to be relatively clear, not too abstract and their interpretation was not too ambiguous. For this reason, we avoided theories of epiphenomenalism (Jackson, 1982) and functionalism (Putnam, 1960; Putnam and Castaneda, 1967), which could be interpreted by the participants in various different ways. For similar reasons, we excluded theories that would seriously confuse the participants, such as parallelism, interactionism, solipsism, neutral monism, panpsychism, and transcendental monism (e.g., Naude, 2009).

The name of the specific mind-body theory was hidden from the participants, only a reasoning of the theory was displayed. The participants were invited to read the argumentation of the specific theory. Later they were asked: *Would you agree with the above theory?* Two answers were presented: *Yes* and *No*. The participants were allowed to choose from only one of these answers. If the participant chose *Yes*, he or she proceeded to another theory. If the participant chose the answer *No*, two additional ideas were presented and he or she could choose only one of them. This method of answering guaranteed that the participants were always offered an option that best suited their intuition (see **Table 5** for a detailed explanation).

### Order of Theories in MBQ

To immediately induce the participants’ genuine intuitions about the mind-body relationship, we chose to start with three theories that directly represented three possible approaches to reductionism. The first was *substance dualism* – the mind is not reducible to the brain. Followed by *non-reductive materialism* – the mind emerges from the brain but is not reducible to brain processes. The last was *reductive materialism* – the mind is nothing more than the brain and can be completely reduced to neuronal processes. This was the easiest way for the participants to immediately identify their own intuitions about the mind-body problem and followed them throughout the whole survey (see **Table 4** for the order of theories).

### Terminology Used and Selection of Terms

From a methodological point of view, we used many terms in our questionnaire that significantly helped the participants who were not familiar with philosophy, to understand the relation between the mind and the body, and thus to easily identify their own intuition about reductionism. In the case of dualism, we used terminology such as: *the mind is completely different to the brain*, *mental phenomena are not material*, *there cannot be a connection between non-material and material*, etc. Non-reductive materialism was accompanied by statements such as: the *mental domain is real*, *mental phenomena are real*, *mental phenomena are not reducible*, etc. Finally, in the case of *reductive materialism*, we frequently used formulae such as: *the mind is nothing more than the brain*, *the mind is nothing more than material processes*, *the mind can be completely reduced to, can be completely explained by*, etc.

### Evaluation of the Mind-Body Questionnaire

For every answer, the participant gained 1 point. Based on the participant’s answer, the point would be allocated to one of the three categories – *dualism*, *non-reductive materialism*, or *reductive materialism*. In this way, we evaluated each of the participants’ intuition about the mind-body problem and their intuition about reductionism of the mind to brain processes. The evaluation was based on the dominant types of answers, for example, if a participant received 7 points for *reductive materialism* and 3 points for *non-reductive materialism* he or she was categorized as a *reductionist*.

### Different Kinds of Answers for Three Specific Theories

In the case of the three theories, *token identity theory*, *supervenience*, and *eliminative materialism*, we decided to use a different method of answering. *Token identity* and *supervenience* are usually understood as being positions of non-reductive materialism; however, from the argumentation (see Appendix 1 for the entire MBQ) the participant could intuitively understand them from the position of non-reductive materialism or from the position of reductive materialism. Therefore, if the participant agreed with these materialistic theories, we left the decision, whether he or she intuitively understands them as positions of non-reductive materialism or reductive materialism, entirely up to the participant. If the participant agreed with the argumentation of *token identity* or *supervenience* and answered *Yes*, then two additional options were presented and he or she was allowed to choose only one of them. One was the position of non-reductive materialism, while the second was the position of reductive materialism. If the participant disagreed with argumentations of *token identity* theory or *supervenience* then the point would be allocated to dualism.

In the case of eliminative materialism, if the participant answered *Yes* and agreed with the argumentation, then the point was put to reductive materialism. However, if the participant answered *No*, then three additional options were presented instead of two. The first option represented the position of dualism and second option was the position of non-reductive materialism. The third option was again the position of reductive materialism; however, without the key idea of eliminative materialism, which states that terms of folk psychology should be replaced with the dictionary of neuroscience. This way, the participant could still choose the position of reductive materialism while disagreeing with the elimination of folk psychology, which is one of the key ideas of eliminative materialism.

### Unclassifiable and Excluded Participants

We established several conditions that excluded participants from the analysis. A participant was considered unclassifiable (and excluded from the final sample) if they scored an almost equal number of points among the three categories. For example, if a participant (based on their answers) received 3 points for *dualism*, 4 points for *non-reductive materialism* and 3 points for *reductive materialism*, then they were considered unclassifiable. This condition was determined as the condition of 3 – 4 – 3. Another condition that excluded a participant from the study was if the participant scored an equal number of points between two categories. For example, if a participant scored 5 points for dualism and 5 points for reductive materialism, as well as when they scored 5 points for dualism and 5 points for non-reductive materialism, and 5 points for reductive materialism and 5 points for non-reductive materialism.

### Temperament and Character Inventory

Cloninger’s Temperament and Character Inventory – Revised (TCI-R) personality questionnaire (Cloninger et al., 1994) was applied to determine seven personality dimensions from 240 self-descriptive statements. The participants answered these statements with a true/false response to indicate their agreement or disagreement with the statement. The original form of all of the statements and response scales according to the Czechia translation (Koženı and Tišanská, 1998; Preiss et al., 2007) was used in the online questionnaire (10 statements per page). The TCI-R evaluates personality dimensions using four temperament scales [novelty seeking (NS), harm avoidance (HA), reward dependence (RD) and persistence (PS)] and three character scales [self-directedness (SD), cooperativeness (CO) and self-transcendence (ST)]. To perform the statistical analysis and to simplify the interpretation of the results, the direct values of individual dimensions were transformed into *z*-scores according to the normative data (Preiss et al., 2007).

### Statistical Analysis

Fisher’s exact test with a level of significance equal to 0.05 was used for assessing the difference in demographic data between dualists, non-reductive materialists, and reductive materialists. For assessing each dimension of the TCI, we applied an analysis of variance (ANOVA) for three groups (when the Shapiro–Wilk test confirmed the normality of data in each group and Levene’s test did not reject the equality of variances between all groups) or the Kruskal–Wallis test (when any of the above-mentioned assumptions for using ANOVA was rejected). Tukey’s *post hoc* test was then conducted to identify which significantly differed. Logistic regression was performed for analyzing the effect of each temperament or character score on the chance of belonging to the group of dualists or reductive materialists. Pairwise Cohen’s ds were calculated to assess the effect size for comparing the different groups. The data were processed in Microsoft Excel 2013 and all of the statistical analyses were performed in IBM Stata IC 14.

## Results

### Mind-Body Questionnaire

On the basis of the MBQ scoring, 12 participants were assessed as being *dualists*, 68 participants were evaluated as being *non-reductive materialists*, and 36 as *reductive materialists.* The remaining 13 participants were excluded from the study based on their unclassifiability (see the section “Unclassifiable and Excluded Participants”). None of the participants of our study scored 5 points for *dualism* and 5 points for *reductionism*).

### The Influence of Demographic Parameters on the Degree of Reduction of the Mind to Physical Brain Processes

For the 116 participants who finished the MBQ, we analyzed their demographic data, describing their gender, relationship, interest in philosophy, and interest in science. In these demographics, dualists, non-reductive materialists, and reductive materialists did not show any significant differences.

Significant differences at a level of 0.05 were found in the demography data for categories of the highest attained education (*p* = 0.010), interest in esoteric and spirituality (*p* = 0.004), and religion beliefs (*p* = 0.011). The vast majority of the dualists and reductive materialists had a university education (75 and 78%, respectively), while more than 50% of the non-reductive materialists studied at high school or were postgraduates. A total of 83% of the dualists, 52% of the non-reductive, and 31% of the reductive materialists were interested in esoteric and spirituality. The majority (45%) of dualists considered themselves to be Christians, while this figure was 24% for the non-reductive materialists and only 9% for the reductive materialists. In total, 57% of the non-reductive materialists and 53% of the reductive materialists did not practice any specific religion and 38% of the reductive materialists considered themselves to be atheists. More detailed results for these variables can be found in **Table 1**.

**Table 1:** Demographic data for each category of mind-body problem inclination.

		Mind-body problem inclination			
		Dualists	Non-reductive materialists	Reductive materialists	Total
Education^∗^	Postgraduate	16.7	32.4	11.1	24.1
	High school	0.0	20.6	8.3	14.7
	Higher vocational school	8.3	2.9	2.8	3.4
	Higher education	75.0	44.1	77.8	57.8
Interest in Esoterics^∗^	Yes	83.3	51.5	30.6	48.3
	No	16.7	48.5	69.4	51.7
Religion Beliefs^∗^	Buddhism	0.0	1.6	0.0	0.9
	Christianity	45.4	23.8	8.8	19.8
	I do not practice any specific religion	27.3	57.1	53.0	49.0
	I consider myself as an atheist	18.2	17.5	38.2	22.4
	Taoism	9.1	0.0	0.0	0.9

^∗^*p-value of Fisher’s exact test lower than 0.05.*

### The Influence of Personality Characteristics on the Degree of Reduction of the Mind to Physical Brain Processes

**FIGURE 1:**
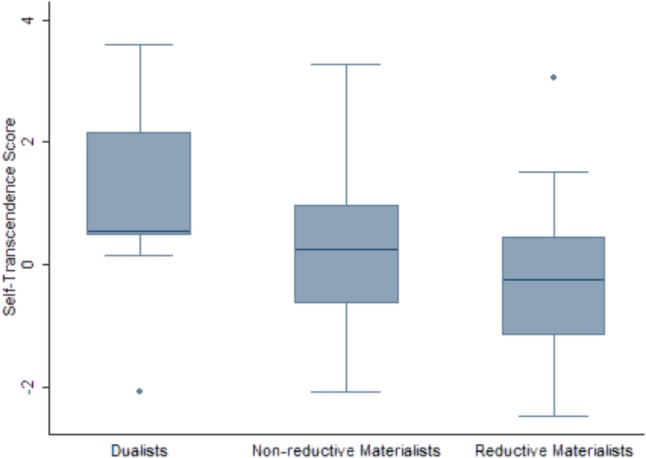
Box plots of self-transcendence score for groups of dualists (*n* = 9), non-reductive materialists (*n* = 53), and reductive materialists (*n* = 32).

In total, 53 of the 94 participants who completed the TCI were non-reductive materialists, 32 were reductive materialists and 9 were dualists. Analysis of the TCI showed that only the self-transcendence (ST) score significantly differed among the *dualists*, *non-reductive materialists*, and *reductive materialists.* The means of the ST scores for these groups were 0.99, 0.33, and -0.27, respectively (**Figure 1** and **Tables 2**, **3**). We also assessed the effect of the ST score on the chance of belonging to the group of *dualists* or *reductive materialists* by logistic regression, which confirmed a significant difference in ST between these two groups.

**Table 2:** Comparison of the three groups of mind-body inclinations: means of all personality dimensions and results of tests.

	Mean			Statistical test	*p*-value
Dimension of personality	Dualists	Non-reductive materialists	Reductive materialists		
Self-Transcendence	0.99	0.33	-0.27	ANOVA	0.023
Novelty Seeking	0.07	0.23	-0.15	ANOVA	0.288
Harm Avoidance	0.00	-0.19	-0.04	ANOVA	0.764
Reward Dependence	0.23	0.00	-0.13	Kruskal–Wallis	0.655
Persistence	0.26	0.52	0.45	Kruskal–Wallis	0.751
Self-Directedness	-0.19	0.08	-0.09	ANOVA	0.664
Cooperativeness	1.02	0.45	0.61	Kruskal–Wallis	0.193

**Table 3:** Descriptive statistics of self-transcendence within each category of mind-body inclination.

	No. of participants	Mean	SD	Min	Max
All	94	0.189	1.34	-2.48	3.60
Dualists	9	0.987	1.63	-2.08	3.60
Non-reductive materialists	53	0.329	1.30	-2.08	3.28
Reductive materialists	32	-0.269	1.21	-2.48	3.04

**Table 4:** The order of theories in the Mind-Body Questionnaire.

1. Substance dualism
2. Non-reductive materialism
3. Reductive materialism
4. Type identity theory
5. Property dualism
6. Supervenience
7. Anomalous monism
8. Emergentism
9. Token identity theory
10. Eliminative materialism

**Table 5:** An example of description of the mind-body theory (*Substance dualism*) and answering.

*Substance dualism*:

*The mind and brain are two very different things – two completely different substances. The brain has the material form and consists of material parts. The mind does not. The mind cannot be split, and the mind does not have parts. The relationship between the mind and the body (brain) can be explained as the spirit that merely inhabits a complex machine.*
*Would you agree with the above theory?*
*Yes: No:*
(1) *The mind is not another substance. The mind is formed by material processes but the mind has qualities that cannot be reduced to the processes of the brain. (Non-reductive materialism)*
(2) *All mental states are in fact solely material processes of the brain. What we now understand as the mind will be in the future completely reducible to the neural processes. (Reductive materialism)*
At the beginning of the questionnaire, the participants immediately encountered *substance dualism* as the first mind-body theory (the name of the theory was hidden from the participants). After reading, the participants were asked whether they would agree or disagree with the argumentation. If a participant chose the answer *Yes:* he or she would acquire one point for *dualistic inclinations* and would proceed to the next theory [which was *non-reductive materialism* (see **Table 4** for the order of theories)]. However, if a participant disagreed with the implications of the theory and therefore answered *No:* two additional options (arguments) were presented to them – in this case, *non-reductive materialism* and *reductive materialism*. From these two additional options, they could choose only one, thus obtaining one point for either *non-reductive materialism inclinations* or *reductive inclinations*.

As the Shapiro–Wilk test did not reject the hypothesis about normality of data within each group (*W* = 0.95–0.97, *p* = 0.290–0.711) and Levene’s test did not reject the equality of variances between the groups (*F* = 0.72, *p* = 0.724), we applied ANOVA, which showed that the *dualists*, *non-reductive materialists*, and *reductive materialists* significantly differed when comparing their ST scores (*F* = 3.96, *p* = 0.023). Tukey’s *post hoc* test was then conducted to identify the groups that significantly differed. Significant differences were found between the dualists and reductive materialists (contrast = -1.25, *p* = 0.033). The effect size was 0.96 (CI = 0.19;1.73) for the difference between the dualists and reductive materialists, 0.49 (CI = 0.02;0.91) and 0.47 (CI = -0.23;1.20) when comparing the non-reductive materialists with the dualists and the reductive materialists with the non-reductive materialists, respectively.

We applied logistic regression to confirm the mediating role of the ST parameter in forming a dualistic or reductive materialistic position. This model showed that one point on the ST scale significantly increased the chance of belonging to the group of dualists, more than twice [OR = 2.02, CI = (1.08;3.72), *p* = 0.028].

The remaining character dimensions, self-directedness, and cooperativeness, as well as all of the temperament dimensions, novelty seeking, harm avoidance, reward dependence, persistence, showed no significant differences.

### Emergence Theory of Mind

As expected, the most favorable and agreeable theory was *Emergentism.* According to our MBQ, 100 (86,2%) of the 116 participants agreed with the argumentation of *Emergentism*.

## Discussion

The main finding of our pilot study is that personality features correlate with intuitions of the mind-body problem and inclination toward a dualistic or reductionist position. The participants who were evaluated as dualists (on the basis of MBQ) scored high positive values in ST, while the participants evaluated as reductive materialists showed a negative ST score. Moreover, the ST values of non-reductive materialists put them into a middle position between the dualists and reductionists.

Even though the dimension of ST was central for the main hypotheses of our study, we also explored whether other personality dimensions would show significant differences among the dualists and reductionists. In our results, none of these two remaining dimensions of character showed any significant difference between the dualists and reductionists, the same as the other four dimensions that represent temperament.

The fact that none of other personality dimensions showed any significant differences between the dualists, non-reductionists and reductionists, indicates the importance of ST as a specific feature of personality. This is in line with the reasons why TCI (Cloninger et al., 1993) replaced the former Cloninger’s Tridimensional Personality Questionnaire (Cloninger et al., 1991). The idea was that the individual need for transcendence or spirituality represents an independent personality feature. Cloninger (2009) assumes that consciousness and self-awareness (of Homo sapiens) developed along the capacities and abilities for spirituality and sense of transcendence. Human consciousness thus includes a unified framework for personality, physicality, emotionality, cognition, and spirituality. Hence, the TCI was the first major theory of personality, which incorporated a spiritual dimension as a core component of personality and not only as the aspect of human functioning (e.g., Taylor and MacDonald, 1999).

Our findings from the TCI are also supported by the results of religiosity from the demographical part of the questionnaire, but they should not be interpreted in the sense that religious beliefs automatically imply the dualistic position. We tested whether those who declared any religious beliefs were more likely to form a dualistic position than those who considered themselves as atheists. No significant difference in the proportion of atheists in the dualists and in the reductive materialists was found (*p*-value for Fisher’s exact test = 0.292) and the binary logistic regression model for forming a dualistic position did not present any results supporting the relationship between having religious beliefs and forming a dualistic position (*p*-value = 0.221). This suggests that mind-body problem intuitions cannot be primarily connected to religious beliefs or atheism. However, due to several limitations of this pilot study (particularly the small number of participants), this conclusion should be taken with caution and it should also be considered that our data are only correlative in nature, i.e., we cannot answer the direction of the causal link between ST, mind-body intuitions and inclinations to religiosity.

According to Cloninger et al. (1999), ST could be a predictive tool for the emergence and subclinical form of mood and psychotic disorder. Our data indicate that the dimension of ST also influences beliefs and intuitions about such theoretical questions as inclination toward a specific category of the mind-body relation and a degree of reduction of the mind to physical brain processes. Further studies should address whether ST also modifies the probability of agreement or disagreement with conclusions of thought experiments of consciousness, such as knowledge argument (Jackson, 1982), what is it like to be a bat (Nagel, 1974), or philosophical zombies (Chalmers, 1996). It could be expected that those who score high on ST (which according to this study is a feature of dualists) would most likely agree with the argumentations and conclusions of thought experiments that advocate irreducibility of consciousness. On the other hand, those who would have a negative ST score (inclination toward reductionism) would most likely disagree with such thought experiments. Another possible research hypothesis can be based on the findings that ST correlates substantially with the NEO-FFI dimension of ‘Openness (O)’ and moderately with ‘Extraversion (E),’ in particular with items O3 (Feelings), O2 (Aesthetics), and E6 (Positive emotions) (De Fruyt et al., 2000). Hence, one can speculate that subjects who are more sensitive and positively tuned incline to the dualistic position. This hypothesis should be addressed by future studies using the NEO-FFI and MBQ.

Interestingly, most participants of our online study inclined to the position of non-reductive materialism, and the most agreeable theory was *Emergentism*. This finding is in line with our original expectation. We hypothesized that this could resemble the development of analytic philosophy of the mind during the past six decades, when it experienced an explosive increase of materialistic conceptions of the mind. At the beginning, it was dominated by reductive materialism, which claimed that any mental type is identical (identity theory) to some physical types (Feigl, 1958; Smart, 1959; Armstrong, 1968). However, reductive materialism faced severe criticisms (e.g., Fodor, 1974) and reductively oriented ideas were soon challenged by the theories of non-reductive materialism, such as anomalous monism (Davidson, 2001), functionalism (Putnam, 1960; Putnam and Castaneda, 1967), and emergentism (e.g., Searle, 1984). It is highly unlikely that the intuitions of the participants, who were not trained in philosophy, are based on profound and sophisticated argumentation against reductionism. So the question is, what drives these intuitive inclinations toward non-reductive materialism and emergentism?

We believe that intuitive positions toward non-reductive materialism and emergentism (*mental sphere emerged from the complexity of the brain, as its new property, which surpasses the features of neurons*) are caused by the fact that non-reductive materialism still leaves the mind its own reality, existence, and volition (Barušs, 2008). According to Barušs (2008), almost all connotations of consciousness entail the agency and volitional aspects. The contrary idea that the mind could be completely reduced to the physical and be completely explained and identified within material, brings with it the disturbing idea that all of our most intimate states of mind and volition are actually not existent. There is a fear that comes with reduction, fear that we are nothing more than not-understanding unconscious robots (Searle, 1980; Dennett, 1987), zombies (Chalmers, 1996), ‘protein robots,’ and ‘mere things’ (Dennett, 2003). The idea that the mind is nothing more than the physical body brings with it the possible loss of the self, volition, and all of the intimate mental phenomena that define it.

“If, in short, there is a community of computers living in my head, there had also better be somebody who is in charge; and, by God, it had better be me” (Fodor, 1998, p. 207) or “Of course the problem here is with the claim that consciousness is ‘identical’ to physical brain states. The more Dennett et al. try to explain to me what they mean by this, the more convinced I become that what they really mean is that consciousness does not exist” (Wright, 2001, fn. 14, ch. 21).

That is why we believe that the intuitive position of non-reductive materialism is much more appealing for people, even without profound philosophical argumentation. The brain creates the mind; however, the mind is so intimate and represents something special, that the person does not want to accept that it would be merely a movement of selfless or mindless molecules. Therefore, we assume that it is much more appealing to think about the brain as a generator of some new property, the mind, which is something new, something more, which little exceeds its physical basis. This assumption is indirectly supported by our finding that the ST feature is more intensively expressed in non-reductive than in reductive materialists, in whom this parameter was even negative (0.30 vs. -0.27). We speculate that the transpersonal component of ST could propagate also to the belief that the mind is something more than a brain functioning.

The intuitive ‘fear of losing mind/consciousness’ could be further studied in the future by experimental philosophy. An experiment could be built on the difference between intuitions about reductionism among scientific disciplines (would you agree that chemistry is completely reducible to physics?) and reductionism concerning the mind (would you agree that the mind is reducible to molecular neuroscience?). It is quite easy to think about science in the context of reductionism, but reductionism tends to drop out when it starts to concern the phenomenal qualities of the human mind.

Based on our findings, the TCI dimension of ST correlates with intuitions of the mind-body problem and intuitions of reductionism. The dimension of ST could be used as a predictive tool for determining inclination toward dualism, non-reductive materialism, or reductive materialism. This finding that intuitions about the mind-body problem have a close connection to the psychological dimension of character is fully congruent with the main goal of contemporary experimental philosophy:

“What we really want to know is why people have the intuitions they do” (Knobe and Nichols, 2008).

However, it would be exaggerated to claim that only the dimension of ST drives intuitions. We believe that dualistic or reductive intuitions permeate the whole character. Nevertheless, ST should be taken into account in future studies on reductionism and the mind-body problem.

There are several limitations of this study. The main limitation is the small sample of participants, which was caused by the scale and the difficulty of the MBQ and by the length of the TCI. Due to the small sample of participants, our results should be considered as preliminary and cannot be used for the universal and clear distinction between dualists, non-reductive materialists, and reductive materialists. However, for the pilot study of this type, the lower number of respondents was expected. Another important limitation is the fact that our MBQ has not been validated. It is the first study of its type and therefore it is impossible to compare our results with other surveys. Nevertheless, the identified association between the dualistic position in MBQ and ST, religiosity and spirituality is to some extent obvious and supports the usefulness of the MBQ approach for future studies. Another support for the MBQ is that the participants evaluated as non-reductive materialists (based on the MBQ) were put between the dualists and reductive materialists based solely on their ST score (**Figure 1**).

Another limitation is related to the issue of the short descriptions of mind-body theories, which do not completely capture the depth of the argumentation of the specific theories. However, this was necessary for two reasons. First, the use of short descriptions was necessary to avoid the participants losing interest in the MBQ. Second, we were more interested in the inner intuitions of the participants about the reduction of the mind to the brain and their position in the categories of dualism, non-reductive, and reductive materialism, rather than ensuring an exhaustive description of the specific mind-body theories. That is also why we changed the method of answering in the case of *token identity theory*, *supervenience*, and *eliminative materialism*, to extract the participants’ intuitions about reduction in the best possible way.

With this in mind, our pilot study should be taken with caution and should be considered as a starting point for future investigations of intuitions of the mind-body problem and intuitions of the reductionism.

## Conclusion

This pilot study showed the close relationship between the dimension of ST and inclination toward a dualist or a reductive position. We also confirmed that the majority of participants inclined toward non-reductive materialism with emergentism as the most agreeable theory of the mind-body relation. This study never aspired to solve the mind-body problem. Instead, this study aimed to identify the factors (features of personality) that play a role in inclination toward dualism or reductionism. Even though studies of this type cannot solve the mind-body problem, finding psychological processes behind such mind-body intuitions would still be a significant step forward in discussions of the philosophy of the mind. Maybe it is time to move away from the solution of the mind-body problem, and put our efforts into the ideas: why is the mind-body problem such a big problem in the first place? Why are there so many different intuitions about it, and what psychological processes distinguish dualists from reductionists?

## Author Contributions

MH wrote the article. KM was responsible for the statistics. IF was responsible for the online survey. JH supervised the study and wrote the article.

## Conflict of Interest Statement

The authors declare that the research was conducted in the absence of any commercial or financial relationships that could be construed as a potential conflict of interest.
