# Supplementary material for: Do Personality Features Influence Our Intuitions of the Mind-Body Problem? A Pilot Study
Source: Front Psychol. 2018 Jul 20;9:1219. doi: 10.3389/fpsyg.2018.01219 (PMC6062648; doi:10.3389/fpsyg.2018.01219)
Supplement: Supplementary file 1 [file Presentation_1.PDF]

Appendix

Mind-Body Questionnaire

1) ***Substance Dualism***: The mind and brain are two very different things - two completely different substances. The brain has a material form and consists of material parts. The mind does not. The mind cannot be split, the mind does not have parts. The relationship between the mind and the body (brain) can be explained as the spirit that merely inhabits a complex machine.
   Would you agree with the above theory?
   Yes (Dualism) / *No*

   If No:
   a) The mind is not another substance. The mind is formed by material processes, but the mind has qualities that cannot be reduced to the processes of the brain. (Non-reductive materialism)
   b) All mental states are in fact solely material processes of the brain. What we now understand as the mind will be in the future completely reducible to the neural processes. (Reductive materialism)

2) ***Non-reductive materialism***: All mental phenomena such as consciousness, emotions, mental states, etc. are real, while the mental domain comes from/emerges from the brain and its processes. However, this relationship between the mind and the brain does not mean that the mental domain is reducible to simple neuronal or physical processes. The mental domain has its own autonomy.
   Would you agree with the above theory?
   Yes (Non-reductive materialism) / *No*

   If No:
   a) Of course, there is a relationship with the brain, but we can expect that the mental domain will be completely transformed into neuronal mechanisms in the future. (Reductive materialism)
   b) The mind and the mental sphere are from the brain and its processes are completely different. (Dualism)

3) ***Reductive materialism***: The relationship between the mental domain and brain is not complex at all. They are the same. It is the relationship of identity. All psychical processes are identical to neural processes. Whole the domain of mental phenomena is nothing else than specifically ordered neural activity and all mental phenomena are/will be fully reducible to material processes.
   Would you agree with the above theory?
   Yes (Reductive materialism) / *No*

   If No:
   a) The mind is completely different, it is something immaterial and completely different from brain processes. (Dualism)
   b) The mind emerges from the brain. Mental states are real and not fully reducible to neural processes. (Non-reductive materialism)

4) ***Type identity theory***: Mental states are nothing more than states of the brain. In this case, we talk about the identity between mental and physical (neuronal) domains. To have various mental states is nothing more than to have specific neural activity. It is possible to identify the whole types of mental processes (e.g. emotions, sensations, beliefs) with types of physical (neuronal) processes (e.g. functionally

anatomical localization of emotion, sensation, etc.). For any pain felt, the same type of neurophysiological mechanisms would always be responsible.
Would you agree with the above theory?
Yes (Reductive materialism) / *No*

If No:
a) It is impossible to speak of identity because there is no possible identity between material and non-material. (Dualism)
b) Some mental states are identical to neural processes, but the mind is autonomous and non-reducible to specific brain processes. (Non-reductive materialism)

5) ***Property dualism***: It is not necessary to introduce two different substances in order to explain the relationship between the mind and the brain. Restricting ourselves to only material substance is sufficient. This material substance however, can take a variety of characteristics such as the mind and mental states, which are not retroactively explainable and reducible to material substance.
Would you agree with the above theory?
Yes (Non-reductive materialism) / *No*

If No:
a) The mind represents a completely different substance. Non-material substance. (Dualism)
b) The mind comes from the brain and in the future it can be expected that all its qualities will be expressed and explained by neural processes. (Reductive materialism)

6) ***Supervenience***: The relationship between the mind and the brain can be described as a relationship based on the necessary dependence of a higher level on a lower level. The mind, representing a higher level, is completely determined by its material basis. In this case, it is not possible for a higher level to remain unchanged if the lower level changes.
Would you agree with the above theory?
Yes / *No*

If Yes:
a) The mind as a higher level of the system can be fully understood by studying the lower levels. (Reductive materialism)
b) Mental states are created by lower levels, but their status is already autonomous and therefore they cannot be converted into mere neural processes representing a lower level. (Non-reductive materialism)

If No:

a) The mind is not a higher level or higher quality of the brain, because it is completely different from the material basis.

7) ***Anomalous monism***: To explain the mind it is not necessary to postulate two different substances. Mental states are essentially states of the brain, neural states or physical states. However, it is not possible to reduce the mental domain to the material domain, because that would require the existence of strict psychophysical laws, which would identify mental states with neuronal states. These strict psychophysical laws will never be formulated and they will not exist.
Would you agree with the above theory?
Yes (Non-reductive materialism) / *No*

If No:

a) Psychophysical laws do not exist because the mind is simply something other than the material brain. (Dualism)
b) Strict psychophysical laws certainly exist. The only thing that is needed is to discover them and formulate them in detail. (Reductive materialism)

8) ***Emergentism***: The mental sphere emerged from the complexity of the brain, as its new property. Thus, the mental sphere acts as a macro-property of the brain, arising from the interaction of neurons. Neurons themselves do not have these qualities (consciousness, mind, emotions) in a similar way as one molecule of water is not liquid. This new macro-property is, therefore, very difficult to explain by the various elements that shape it.
Would you agree with the above theory?
Yes (Non-reductive materialism) / *No*

If No:

a) The mind is not a macro-property of the brain, because it is a completely different substance. (Dualism)
b) The mind is not difficult to fully explain from brain processes; however, we still do not yet have detailed measurements and we do not know the neural processes in sufficient detail. (Reductive materialism)

9) ***Token identity theory***: The relationship between the mind and the brain is a relationship of identity. All mental states are identical to states of the brain, and on this basis it would be possible to identify specific mental processes with specific neurophysiological processes.
Would you agree with the above theory?
Yes / *No*

If Yes:

a) Identity is a suitable term to describe the relationship between the mind and the brain, but in no way should imply that mental states are nothing but brain states. (Non-reductive materialism)
b) Identity is an appropriate term to describe the relationship between the mind and the brain; however, it should explicitly claim that mental states are nothing but brain states. (Reductive materialism)

If No:

a) The specific material states of the brain cannot be identical to specific non-material states of the mind. (Dualism)

10) ***Eliminative materialism***: It is unnecessary to talk deeply about the relationship between the mind and the brain, because merely talking about mental states is misleading. Finding the relationship between the mind and the brain in a way that will reduce the mind to the brain itself requires prior knowledge of what should be reduced. However, nothing like a mental states exist and therefore it is rational not to reduce mental states to physical, but directly replace our dictionary terms of folk psychology with a dictionary of neuroscience.
Would you agree with the above theory?
Yes (Reductive materialism) / *No*

If No:

a) The mind truly cannot be reduced to the brain, but not because the mental does not exist, but because the mental states represent a totally different substance. (Dualism)
b) A strong relationship between the mind and the brain is unquestionable, but mental terms cannot be replaced by neurophysiological terms, since the mind itself is an autonomous sphere. (Non-reductive materialism)
c) Of course, we will be able to reduce the mind to specific processes of the brain, but that does not mean that we must replace (and essentially eliminate) our mental concepts with neuroscience terms (Reductive materialism).
